# Awareness of Diabetic Retinopathy Among Patients With Diabetes Mellitus in the Ernakulam District, South India: A Hospital-Based Cross-Sectional Study

**DOI:** 10.7759/cureus.71414

**Published:** 2024-10-14

**Authors:** Liji Menon M, Neeraj V Mohandas, Tara Susan Mohan, Neethu George, Vinod Mohandas, Malavika C K, Saji Subramanian

**Affiliations:** 1 Ophthalmology, Malankara Orthodox Syrian Church (MOSC) Medical College Hospital, Kochi, IND; 2 Community Medicine, Dhanalakshmi Srinivasan Medical College and Hospital, Perambalur, IND; 3 General Surgery, Hafar Al-Batin Central Hospital, Hafar Al-Batin, SAU; 4 Cardiology, Samaritan Heart Institute, Kochi, IND

**Keywords:** diabetes, diabetic retinopathy (dr), knowledge, ocular complications, retinal complications

## Abstract

Introduction

India is currently experiencing a significant burden of diabetes mellitus, characterized by its high prevalence and associated complications. Diabetic retinopathy (DR) is a major microvascular complication of diabetes, leading to blindness. Awareness regarding this ocular complication of diabetes can help prevent vision loss due to early screening and diagnosis. However, awareness of DR among diabetes patients remains low, especially among developing nations, which affects the smooth functioning of health programs and interventions. This study aimed to determine the independent predictors of awareness regarding DR among patients with diabetes mellitus in the Ernakulam district of Kerala state in South India.

Methods

A hospital-based cross-sectional study was conducted from June 2024 to July 2024 among patients with diabetes mellitus attending a tertiary care hospital in Kerala. A pre-tested, structured questionnaire was used to collect the data. The patients’ responses regarding awareness of DR were scored on a two-point scale, with correct responses receiving a score of 2 and incorrect responses receiving a score of 1. The total score ranged from 20 to 40. The data were entered into Microsoft Excel (Microsoft® Corp., Redmond, WA, USA), numerically coded, and analyzed using IBM SPSS Statistics for Windows, Version 26 (Released 2019; IBM Corp., Armonk, NY, USA). Descriptive analysis was carried out to characterize the study participants and was expressed in frequencies, percentages, and mean (±standard deviation (SD)). Independent samples t-test and one-way analysis of variance (ANOVA) were used for bivariate analyses. A multivariable linear regression model was used to determine the independent predictors of DR awareness score. A p-value <0.05 was considered statistically significant.

Results

The study included a total of 253 patients. The mean ± SD age of the study participants was 58.74 ± 12.51 years, and the majority (55.7%) were females. While 161 (63.6%, 95% CI: 57.71-69.56) patients were not aware that DR was due to the abnormal changes in the blood vessels of the retina, 219 (86.6%, 95% CI: 82.36-90.76) patients were aware that DR screening includes evaluation of the retina by dilating the eye. The independent predictors that had a positive impact on DR awareness scores were: (1) age (B = 1.46; 95% CI: 1.18-2.35), (2) education (B = 4.32; 95% CI: 3.57-5.06), and (3) family history of diabetes (B = 1.04; 95% CI: -1.79 to -0.29). The independent predictor that had a negative impact on DR awareness scores was the occupation of the patients: (a) semi-professional, skilled, unskilled (B = -1.24; 95% CI: -2.26 to -0.21), and (b) unemployed, retired (B = -1.32; 95% CI: -2.43 to -0.21).

Conclusion

It is crucial to evaluate the awareness of DR among patients with diabetes in the Ernakulam district of Kerala, South India. Age, graduate and post-graduate levels of education, as well as family history of diabetes, were the positive predictors of awareness, while lower-level occupations, including unemployment and retirement, were the negative predictors. This information is crucial for developing effective strategies for the early detection, treatment, and prevention of this ocular complication of diabetes. Future research should include longitudinal studies coupled with in-depth interviews, which would provide valuable insights into public perception and attitudes toward DR.

## Introduction

India's emergence as a global epicenter of the diabetes mellitus pandemic is attributable to a confluence of socioeconomic factors, demographic shifts, and genetic predispositions [1]. While the prevalence of diabetes has surged in India over the past four decades, inadequate awareness, a dearth of healthcare professionals, and the prohibitive cost of essential medications and services impede the effective management of this non-communicable disease (NCD) [2].

Asian Indians, comprising over 17% of the world’s population, have a unique phenotype characterized by high levels of intra-abdominal fat and insulin resistance despite low BMI, predisposing them to early-onset diabetes mellitus and its complications [3]. The prevalence of diabetes, as per the Indian Council of Medical Research-India Diabetes-17 (ICMR-INDIAB-17) study, is 11.4% in India and 23.6% in the state of Kerala [4]. The microvascular and macrovascular complications of diabetes mellitus account for the majority of the disease’s morbidity and mortality. While poor glycaemic control and long disease duration are significant factors, ethnic differences in susceptibility to complications may also play a role [2].

Diabetic retinopathy (DR), a leading cause of avoidable blindness, is the most common microvascular complication of diabetes [1]. A recent meta-analysis conducted by Brar et al. reported that the prevalence of DR in India was 17.44% in urban areas and 14% in rural areas [5]. Similar figures were reported by other studies across India, which also report that the risk factors for developing DR were the duration of diabetes mellitus and poor glycaemic control [6,7]. Gurudas et al. in 2024 reported that the prevalence of vision impairment and blindness due to DR in India is 21.1% and 2.4%, respectively [8]. About one‑fifth of known diabetics are projected to have DR, which has a chronic course with a long latent phase [1].

Early intervention, including screening and treatment in patients with diabetes, can prevent a significant portion of DR-related visual impairment. Treatment interventions at the early stages of DR can significantly reduce the burden of blindness due to DR. With the available cost‑effective screening methods, appropriate strategies can be utilized for the early diagnosis of DR. Enhanced awareness can motivate patients to invest more time and resources in seeking a diagnosis [9]. Hence, raising awareness among patients with diabetes, their families, and high-risk individuals is essential for promoting the early diagnosis and screening of DR [10].

However, the lack of dilated fundus examinations in routine surveys conducted across India has limited our understanding of the epidemiology of DR [11]. Implementing an integrated DR screening and management program within India’s healthcare system is crucial [12]. The National Program for Control of Blindness (NPCB) of India currently relies only on opportunistic screening of DR in high-risk populations, which emphasizes early diagnosis, referral, and management at every possible point of contact between the patient and the healthcare provider [10].

Hence, early screening for avoidable ocular complications of diabetes, such as DR, is essential in addition to the timely diagnosis and treatment of diabetes mellitus. This would better assist in long-term care management and improve the health and wellness of the patients [13]. Early screening and diagnosis can only be improved by increasing awareness of this ocular complication of diabetes among patients. This hospital-based cross-sectional study was conducted in the Ernakulam district of Kerala, South India, to determine the independent predictors of awareness regarding DR among patients with diabetes mellitus.

## Materials and methods

Study setting

A hospital-based cross-sectional study was conducted among patients with diabetes mellitus attending the Ophthalmology Outpatient Department (OPD) of a tertiary care center (Malankara Orthodox Syrian Church (MOSC) Medical College Hospital) in Ernakulam District of Kerala state, South India, for a period of two months, from June 2024 to July 2024. Ethical clearance (MOSC/IEC/614/2022) was obtained from the Institutional Ethical Committee of Malankara Orthodox Syrian Church (MOSC) Medical College Hospital prior to the commencement of the study.

Diagnostic criteria

Diagnosis of diabetes mellitus was based on the diagnostic criteria given by the ICMR [14]. Only those patients who were medically diagnosed with Type I and Type II diabetes mellitus using the above-mentioned criteria were included in the study after verifying their medical records. Exclusion criteria: (1) patients who were already previously diagnosed with DR, (2) patients diagnosed with only impaired fasting glucose or impaired glucose tolerance, (3) children aged less than 12 years, and (4) patients with incomplete medical records.

Sample size determination

The sample size was calculated from the proportion of patients who had adequate awareness regarding DR in Goa, India (34.1%), from the study by Venugopal et al. [15]. The formula used was n = Z²(1 - α/2) PQ/d² (Z(1 - α/2) = 1.96, P (prevalence) = 34.1, Q (100 - prevalence) = 65.9, d (absolute precision) = 6%), and the minimum sample size, with 80% power and a 95% confidence interval, came up to 240. However, 253 patients were enrolled in the study for final analysis.

Sampling technique and data collection

A convenient sampling technique was used to collect the data from the patients who attended the Ophthalmology OPD by interview method. A pre-tested, structured questionnaire was used to collect the data. The first version of the questionnaire was developed after consulting an expert panel, and it was pilot-tested among 20 patients who were later excluded from the analysis. The modifications made after the pilot testing were incorporated into the final questionnaire. The internal consistency of the questionnaire was checked using Cronbach’s alpha, and a score of 0.69 was found to be acceptable. The first section of the questionnaire included the details of sociodemographic characteristics, duration of diabetes, and history of other co-morbidities. The second section included a set of 20 questions, which was utilized to assess patient awareness of DR and their inclination to invest further in its diagnosis.

Statistical analysis

The data collected were entered into Microsoft Excel (Microsoft® Corp., Redmond, WA, USA), numerically coded, and analyzed using IBM SPSS Statistics for Windows, Version 26 (Released 2019; IBM Corp., Armonk, NY, USA). The patients’ responses regarding awareness of DR were scored. A “correct response” was given a score of 2, and an “incorrect response” was given a score of 1. The total score ranged from 20 to 40. Descriptive analysis was conducted to characterize the study population and was expressed in frequencies, percentages, as well as mean (±standard deviation (SD)). The Kolmogorov-Smirnov test was used to determine the normality of data. Bivariate analysis was conducted using independent samples t-test and one-way analysis of variance (ANOVA). Pearson’s correlation coefficient was used to assess the correlation of age and duration of diabetes with the DR awareness score. A multivariable linear regression model was applied to determine the independent predictors of awareness regarding DR. All variables with a p-value <0.05 in bivariate analysis were considered for the multivariable linear regression model. The variation in total awareness score due to the independent variables was indicated by the adjusted R-squared value. The Durbin-Watson statistic was used to assess the need for autocorrection. An unstandardized B coefficient was used to predict the change with respect to the dependent variable. A p-value <0.05 was considered statistically significant.

## Results

The mean ± SD age of the 253 study participants was 58.74 ± 12.51 years. Of the study participants, 131 (or 51.8%) were aged less than 60 years, and the majority of the patients were female (141, or 55.7%). The basic characteristics of the study participants are given in Table 1.

**Table 1:** Basic characteristics of the study participants (N = 253)

Basic characteristics		Frequency (n)	Percentage (%)
Age (in years)	≤60	131	51.8
	>60	122	48.2
Gender	Male	112	44.3
	Female	141	55.7
Education	No formal education	6	2.3
	Primary and middle school (1-7 standards)	28	11.1
	High and higher secondary school (8-12 standards)	79	31.2
	Graduate	97	38.4
	Post-graduate	43	17
Occupation	Professional	30	11.9
	Semi-professional	43	17
	Skilled	41	16.2
	Unskilled	62	24.5
	Unemployed	54	21.3
	Retired	23	9.1
Duration of diabetes mellitus (in years)	0-10	185	73.1
	11-20	51	20.2
	>20	17	6.7
Co-morbidities/medical complications	None	77	30.4
	Hypertension	145	57.3
	Other microvascular complications of diabetes (neuropathy, nephropathy)	31	12.3
Family history of diabetes mellitus	Yes	175	69.2
	No	78	30.8

**Figure 1:**
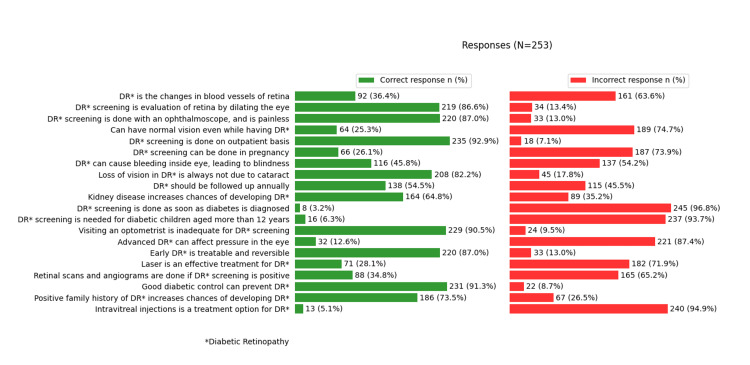
Awareness of the patients regarding diabetic retinopathy (N = 253) *Diabetic retinopathy

While 63.6% (95% CI: 57.71-69.56) were not aware that DR was due to the abnormal changes in the blood vessels of the retina, 86.6% (95% CI: 82.36-90.76) were aware that DR screening includes evaluation of the retina by dilating the eye. The details of the questions, as well as the study participants’ responses, are given in Figure 1.

**Figure 2:**
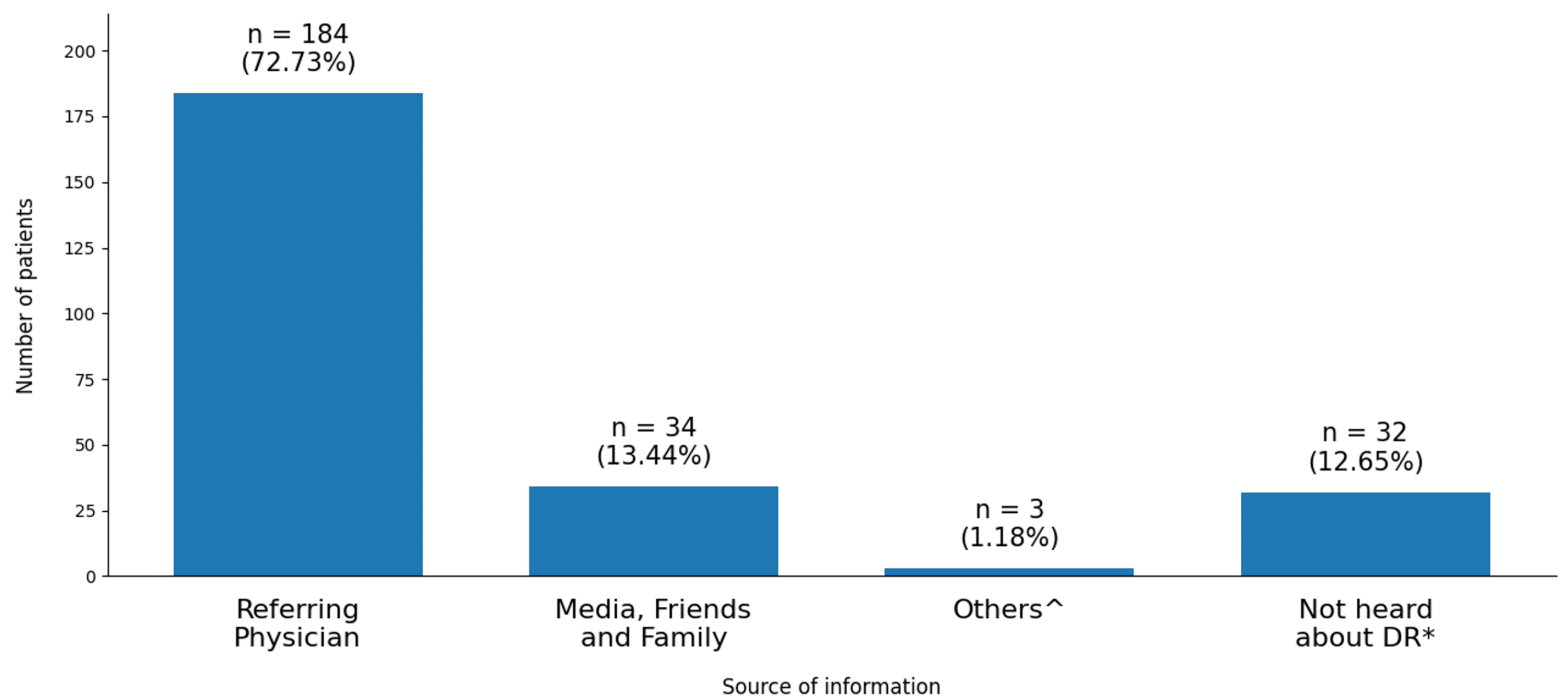
Sources of patient knowledge regarding diabetic retinopathy (N = 253) *Diabetic retinopathy; ^Paramedical staff, community workers, other patients

The primary source of patient knowledge regarding DR was their referring physicians, with 72.73% of the participants learning about the condition from this source. However, a significant minority (12.65%) had no prior awareness of DR. The remaining details are depicted in Figure 2.

**Figure 3:**
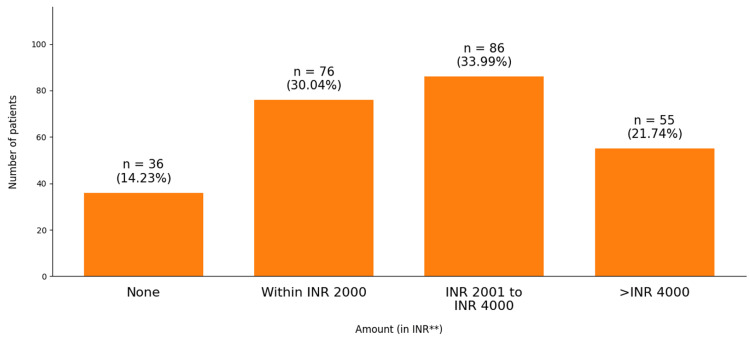
Willingness of the patients to invest in additional tests to diagnose diabetic retinopathy (N = 253) **Indian rupee

As depicted in Figure 3, the majority (33.99%) of the participants were willing to invest between ₹ 2001 and ₹ 4000 for additional tests to diagnose DR, while 30% were willing to spend less than ₹ 2000. However, 21.74% indicated a willingness to spend more than ₹ 4000, and 14.2% expressed no willingness to incur additional costs.

The mean (±SD) DR awareness score among the patients was 30.33 ± 4.29. A statistically significant difference was found between the mean DR awareness scores and the following socio-demographic variables: (1) age (p-value < 0.001), (2) education (p-value < 0.001), (3) family history of diabetes (p-value < 0.001), (4) willingness for additional investment for the diagnosis of DR (p-value < 0.001). The details are given in Table 2.

**Table 2:** Variation of diabetic retinopathy awareness score among patients with diabetes based on socio-demographic characteristics (N = 253) *A p-value of <0.05 is statistically significant Independent samples t-test was used

Basic characteristics		Mean DR awareness score (±SD)	Table value	p-value
Age (in years)	≤60	31.34 (±3.87)	2.69	<0.001*
	>60	29.26 (±4.47)		
Gender	Male	30.75 (±3.97)	2.98	0.17
	Female	30.01 (±4.51)		
Education	Non-formal and formal education	27.00 (±2.97)	0.058	<0.001*
	Graduation and post-graduation	33.03 (±3.15)		
Family history of diabetes	Yes	31.81 (±3.62)	0.13	<0.001*
	No	27.02 (±3.83)		
Willingness for additional investment for the diagnosis of diabetic retinopathy	None	24.11 (±3.34)	-11.62	<0.001*
	Up to and above ₹ 4000	31.37 (±3.48)		

A statistically significant difference was found between the mean DR awareness scores and the following specific variables: (1) current occupation (p-value < 0.001), (2) comorbidities (p-value = 0.042), and (3) sources of information on DR (p-value < 0.001). The details are given in Table 3.

**Table 3:** Variation of diabetic retinopathy awareness score among patients with diabetes based on specific characteristics (N = 253) *A p-value <0.05 is statistically significant One-way analysis of variance (ANOVA) test was used

Basic characteristics		Mean DR awareness score (±SD)		Table value	p-value
Current occupation	Professional	33.16 (±4.57)		9.53	<0.001*
	Semi-professional, skilled, unskilled	30.32 (±4.01)			
	Unemployed, retired	29.25 (±4.25)			
Co-morbidities	None	29.38 (±5.09)		3.21	0.042*
	Hypertension	31.41 (±2.48)			
	Other microvascular complications of diabetes (neuropathy, nephropathy)	30.61 (±4.06)			
Duration of diabetes (in years)	0-10	30.18 (±4.31)		2.52	0.08
	20-Nov	31.37 (±3.78)			
	>20	28.94 (±5.14)			
Sources of information on diabetic retinopathy	Referring physician	31.15 (±3.22)	104.48		<0.001*
	Online and offline media, friends and family	32.72 (±3.48)			
	Not heard about diabetic retinopathy at all	22.87 (±2.51)			

A post hoc Tukey test showed that there is a statistically significant difference in the mean DR awareness scores between the patients: (1) occupation: professional-semiprofessional, skilled, unskilled (mean difference: 2.84); professional-unemployed, retired (mean difference: 3.90), (2) sources of information on DR: online-offline media, friends, family-referring physician (mean difference: 1.57); online-offline media, friends, family-not heard about DR (mean difference: 9.85).

Correlates of DR awareness score

The Pearson correlation revealed a statistically significant positive correlation between the age of the patients and the DR awareness score (r = 0.317, p-value < 0.001). No statistically significant correlation was found between the duration of diabetes and the DR awareness score (r = -0.29, p-value = 0.646).

Regression analysis

The DR awareness score was regressed on predicting variables that were statistically significant in the bivariate analysis (age, education, occupation, family history of diabetes, and co-morbidities). The overall regression model was statistically significant, and the independent variables significantly predicted the DR awareness score, F(10, 242) = 64.94, p-value < 0.001. It showed an R-squared value of 0.558 and an adjusted R-squared value of 0.545, which implies that age, education, occupation, and family history of diabetes accounted for 54.5% of the variation in the DR awareness score. The Durbin-Watson statistic was 2.11, indicating no autocorrelation.

In this study, the statistically significant positive predictors that had an impact on the DR awareness scores were: (1) age (B = 1.46, t-value = 1.40, p-value < 0.001), (2) education of the patients (B = 4.32, t-value = 11.47, p-value < 0.001), (3) family history of diabetes (B = 1.04, t-value = 2.74, p-value = 0.006). The statistically significant negative predictors that had an impact on the DR awareness scores were: occupation of the patients - (a) semi-professional, skilled, and unskilled occupation (B = -1.24, t-value = -2.39, p-value = 0.01), (b) unemployment as well as retirement (B = -1.32, t-value = -2.35, p-value = 0.01). The results are presented in Table 4.

**Table 4:** Independent predictors of diabetic retinopathy awareness score *A p-value <0.05 is statistically significant Multivariable linear regression was used

Variables		Unstandardized B co-efficient (95% CI)	Standardized beta co-efficient	t-value	p-value
Age (in years)		1.46 (1.18 to 2.35)*	0.05	1.40	<0.001*
Education	Non-formal and formal education	1	-	-	-
	Graduation and post-graduation	4.32 (3.57 to 5.06)*	0.50	11.47	<0.001*
Current occupation	Professional	1	-	-	-
	Semi-professional, skilled, unskilled	-1.24 (-2.26 to -0.21)*	-0.14	-2.39	0.01*
	Unemployed, retired	-1.32 (-2.43 to -0.21)*	-0.14	-2.35	0.01*
Family history of diabetes	Yes	1.04 (-1.79 to -0.29)*	-0.11	2.74	0.006*
	No	1	-	-	-
Co-morbidities/medical complications	None	1	-	-	-
	Hypertension	0.78 (-0.29 to 1.86)	0.06	1.43	0.15
	Other microvascular complications of diabetes (neuropathy, nephropathy)	0.68 (-0.02 to 1.38)	0.07	1.91	0.058
Sources of information on diabetic retinopathy	Referring physician	1	-	-	-
	Online and offline media, friends and family	-0.07 (-0.98 to 0.83)	-0.06	-0.16	0.86
	Not heard about diabetic retinopathy at all	-6.88 (-8.49 to -5.27)	-0.53	-8.42	0.45
Willingness for additional investment for the diagnosis of diabetic retinopathy	Up to and above ₹ 4000	1	-	-	-
	None	-1.13 (-2.68 to 0.42)	-0.09	-1.43	0.15

## Discussion

This study provides an overview of the awareness of DR among patients with diabetes mellitus attending a tertiary care center in Ernakulam district of Kerala state, South India. It also throws light on the perception of the patients regarding the disease, as well as their willingness for additional investment in diagnosing this microvascular complication.

In this study, education and occupation of the patients had a significant impact on the awareness of DR. This was consistent with the results of a hospital-based study done in Goa, India, by Venugopal et al., which reported that awareness and knowledge of DR were significantly high among the patients who completed college-level education [15]. These findings reinforce the fact that education, as well as health awareness, always go hand in hand, which is very crucial for the management of chronic diseases. The level of education is always a positive predictor of the health awareness outcome [16]. However, a systematic review done by Suhail et al. has reported that awareness regarding diabetes improves with increasing duration of the disease [17]. Previous studies have used diverse scales and questionnaires to measure the knowledge, attitude, and practices (KAP) of DR, making cross-study comparisons challenging [18,19].

Among those aware of DR in this study, the majority reported obtaining the information from their referring physicians. This finding aligns with the report of a community-based study by Dinesh et al., which revealed that the patients who followed up with professional healthcare providers of modern medicine exhibited enhanced knowledge of diabetes and its complications [20]. The majority of the patients in this study were willing to spend an additional amount between ₹ 2001 and ₹ 4000 to diagnose DR. This can be attributed to the sensitization of the public to timely health interventions in the state of Kerala [21].

In this study, there was a statistically significant and positive impact of age on DR awareness scores. Pardhan et al. have reported that lower KAP scores were significantly associated with age [22]. The finding in this study can also be partially attributed to the experience and awareness gained after multiple visits to the physician over the course of time, as diabetes is a chronic disease. Studies across the world have reported that older age groups have better preventive practices as well as health literacy [23]. This study did not find any statistically significant impact of the duration of diabetes on DR awareness. The majority of the patients in this study had completed either their graduation or post-graduation, which can be partially attributed to the high level of literacy in the state of Kerala [24].

In this study, a family history of diabetes had a statistically significant positive impact on DR awareness scores. A positive family health history is a key predictor of health risk and is universally important in preventive care [25]. Recent studies have shown that patients with a positive family history of diabetes are more prone to early onset of diabetes and developing complications [26]. The high incidence of NCDs, coupled with the high literacy rate in the state of Kerala, could probably be the reason for the positive impact of family history on the awareness of DR [27].

This study employed a rigorous methodology, utilizing a pre-tested questionnaire and advanced regression models to analyze the data. High data consistency was likely, as a dependable interview team was utilized for data collection.

Limitations

Patients attending the Ophthalmology OPD of only a single tertiary care center were considered for this study; hence, the results may not reflect the broader populations in India. This study employed a convenient sampling technique, which may have introduced selection bias. While this study focused on assessing the awareness of DR, it did not include questions related to the patients' understanding and attitudes regarding diabetes mellitus as a disease. The inability to establish causality, due to the cross-sectional nature of this study, is an additional consideration.

## Conclusions

This study underscores the critical need for a comprehensive assessment of the awareness of DR among patients with diabetes in the Ernakulam district in the state of Kerala, South India. The positive predictors of awareness regarding DR included age, graduate and postgraduate levels of education, as well as family history of diabetes, whereas semi-professional, skilled, and unskilled professions, along with unemployment and retirement, were the negative predictors. This knowledge will empower policymakers, healthcare providers, and community groups to develop effective strategies for targeted health education, early diagnosis, treatment, and prevention of DR in the region. Future research should explore public perceptions and attitudes toward this ocular complication of diabetes through longitudinal studies and in-depth interviews.
